# Heterogeneous nuclear ribonucleoprotein A1 as a candidate associated with pulmonary vascular remodeling in pulmonary arterial hypertension

**DOI:** 10.1016/j.jmccpl.2026.100867

**Published:** 2026-08-31

**Authors:** Yoshihito Morimoto, Yoshiaki Sato, Katsuhiro Kato, Hidenori Yamamoto, Kentaro Suzuki, Kiyotaka Go, Yoshie Fukasawa, Naoki Ohashi, Yoshiyuki Takahashi, Taichi Kato

**Affiliations:** a Department of Pediatrics, Nagoya University Graduate School of Medicine, Nagoya, Aichi, Japan; b Department of Pediatric Cardiology, National Cerebral and Cardiovascular Center, Suita, Osaka, Japan; c Division of Neonatology, Center for Maternal-Neonatal Care, Nagoya University Hospital, Nagoya, Aichi, Japan; d Department of Cardiology, Nagoya University Graduate School of Medicine, Nagoya, Aichi, Japan

**Keywords:** Plexiform lesion formation, Unobstructed pulmonary arteries with medial hypertrophy, Differentially expressed proteins, Rat model

## Abstract

**Rationale:**

Although plexiform lesion (PL) formation in severe pulmonary arterial hypertension (PAH) is a therapeutic target, the mechanisms underlying their formation have not been fully elucidated.

**Objective:**

To identify candidate proteins involved in PL formation by examining differentially expressed proteins (DEPs) in PAH lesions.

**Methods:**

Proteomic remodeling was assessed before and after the formation of PLs in a SU5416 combined with hypoxia (SuHx) rat model of severe PAH using laser-capture microdissection coupled with mass spectrometry. Unobstructed pulmonary arteries with medial hypertrophy (UMHPAs) and PLs from SuHx rats were subjected to qualitative and quantitative proteomics, revealing DEPs between these structures.

**Results:**

We identified 718 proteins with 58 DEPs, of which 31 were upregulated in UMHPAs and 27 were upregulated in PLs. Immunostaining confirmed that DEPs detected in our proteomic analysis were differentially expressed between UMHPAs and PLs. Among them, we focused on heterogeneous nuclear ribonucleoprotein A1 (hnRNPA1) as a candidate protein that may be associated with PL formation because of its strong association with cell proliferation. Small interfering RNA knockdown of hnRNPA1 in hypoxia-treated pulmonary artery smooth muscle cells reduced pyruvate kinase M2 expression and decreased proliferative capacity.

**Conclusions:**

Several DEPs associated with PL formation but with unclear relevance to pulmonary artery remodeling in PAH were discovered. Among these, hnRNPA1, which was not detected in transcriptome analysis and whole lung analysis, may be important in PL formation.

## Highlights

• Potential therapeutic targets for pulmonary arterial hypertension are reported.
• We found differential proteins related to plexiform lesions and pulmonary arteries.
• hnRNPA1 may be particularly important for artery remodeling and lesion formation.

## Graphical abstract

**Figure:**
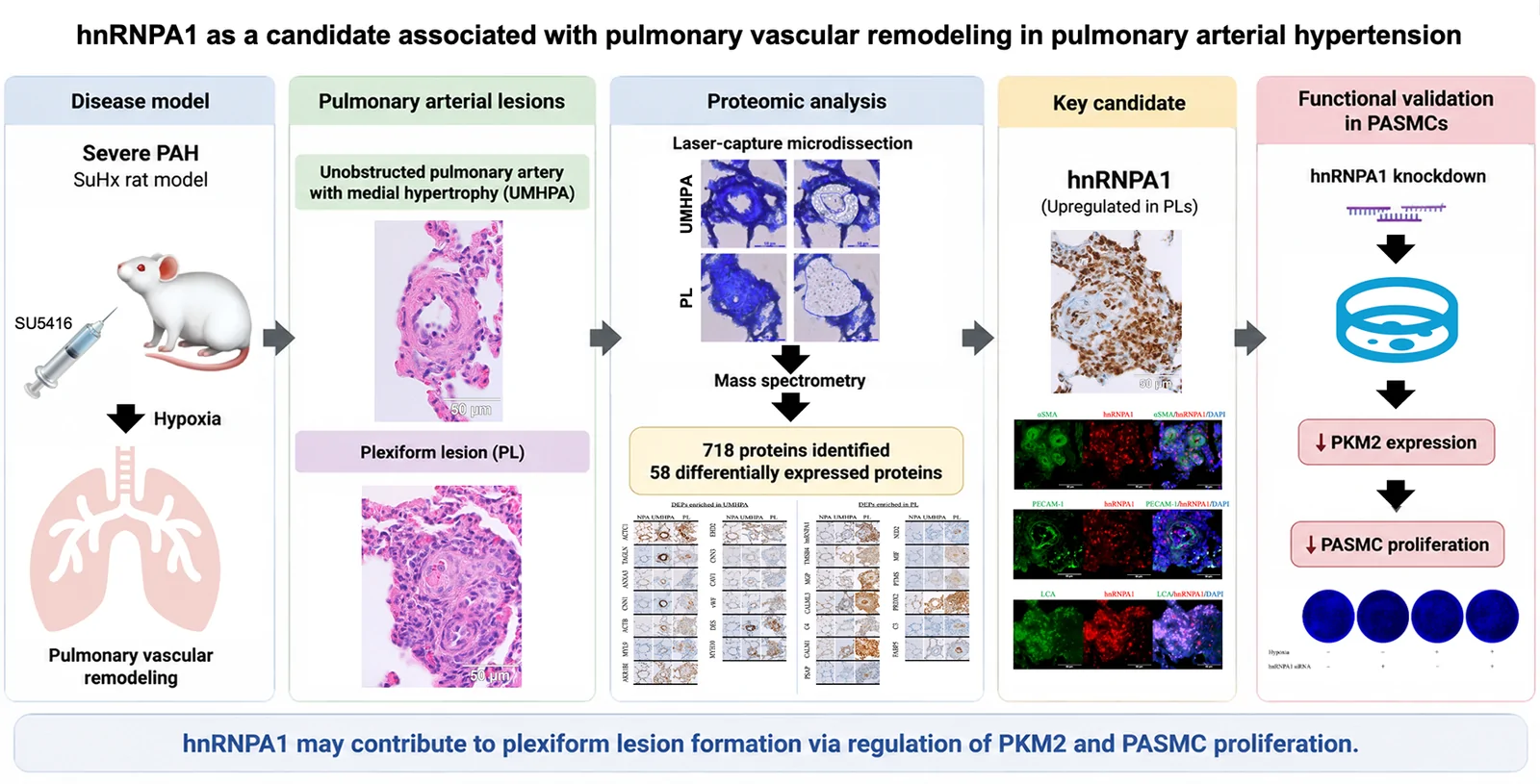

## 1 Introduction

Pulmonary arterial hypertension (PAH) is a progressive, intractable disease characterized by chronic narrowing of the peripheral pulmonary arteries, leading to increased pulmonary artery pressure. PAH, a life-threatening disease, starts with thickening of the tunica media and progresses to intimal proliferation and generation of obstructive lesions. Severe PAH eventually leads to plexiform lesions (PLs) with necrosis of the tunica media and disruption of the vascular structure [1]. PLs are composed of diverse cell types, including pulmonary artery endothelial cells, pulmonary artery smooth muscle cells (PASMCs), fibroblasts, and immune cells. Conventional drugs alleviate lumen narrowing caused by tunica media thickening through pulmonary vasodilation. New promising drugs such as sotatercept (an activin signaling inhibitor) have become available, but the prognosis for severe PAH that forms PLs remains poor [2], [3]. Therefore, identifying therapeutic targets to inhibit PL formation is imperative. However, the mechanisms underlying PL formation remain unclear, largely because PLs coexist with other tissues in the lungs, presenting challenges in analyzing PLs in isolation.

Laser-capture microdissection coupled with mass spectrometry (LCM–MS) is an efficient method for the systematic identification and quantification of proteins in tissue samples. This method can accurately detect regional tissue differences owing to its ability to maintain the integrity of data related to protein localization and spatial relationships [4], [5], [6]. Although proteomic analyses have been performed on whole lungs from PAH patients, containing several other tissues, few LCM–MS procedures have been performed on pulmonary arteries (PAs), and there have been no reports on PLs [7], [8], [9]. This may be because PAs are luminal structures, and obtaining the necessary amount of tissue for LCM–MS is not always straightforward. Similar research methods involving microRNA transcriptomes after LCM for PLs have been reported [2], [10]; however, a substantial discrepancy (approximately 50%) between transcriptome and proteome analysis results has been noted, potentially resulting in divergent conclusions.

Although PLs are therapeutic targets, there have been no direct examinations of proteomic remodeling before and after their formation. Thus, here, we aimed to examine proteomic remodeling using LCM–MS (with formalin-fixed paraffin-embedded (FFPE) sections) before and after PL formation in a rat model of severe PAH involving injection of SU5416, a selective inhibitor of vascular endothelial growth factor receptors, combined with hypoxia (SuHx) [11]. Based on proteome analysis results, we sought to perform a functional analysis of heterogeneous nuclear ribonucleoprotein A1 (hnRNPA1) as a candidate protein involved in PA remodeling in PAH and PLs.

## 2 Methods

### 2.1 Generation of SuHx rat model

All animals received humane care, and all experimental procedures were approved by the Animal Care and Use Committee of Nagoya University (M240320–001 and M240315–001). All procedures conformed to the guidelines from Directive 2010/63/EU of the European Parliament on the protection of animals used for scientific purposes.

We generated a SuHx rat model following the literature [11]. Briefly, adult male Sprague–Dawley rats (Japan SLC, Japan) weighing 180–220 g (*n* = 10) were injected subcutaneously with SU5416 (Cat# 3037, Tocris Bioscience, UK) at a dose of 20 mg/kg, which was suspended in carboxymethylcellulose (0.5% [wt/vol] carboxymethylcellulose sodium, 0.9% [wt/vol] NaCl, 0.4% [vol/vol] polysorbate, 0.9% [vol/vol] benzyl alcohol in deionized water) and were maintained in a hypobaric hypoxic chamber (0.5 atm) for 3 weeks. Thereafter, one group (*n* = 5) was returned to normoxia (21% O_2_) for 5 weeks to induce the middle stage of PAH (8w model), whereas the other group (*n* = 5) was returned to normoxia for 10 weeks to induce the advanced stage of PAH (13w model). The 8w model has many unobstructed pulmonary arteries (PAs) with medial hypertrophy (UMHPAs) without plexiform lesions (PLs), whereas the 13w model has many PLs. Rats received regular chow ad libitum and were housed under a 12:12 h light–dark cycle.

### 2.2 Hemodynamic measurements and sample collection

Rats were placed on controlled heating pads (35 °C) after anesthetization with intraperitoneal pentobarbital sodium (30 mg/kg). Hemodynamic measurements were performed under normoxic conditions. A polyethylene tube (SP-31, Natsume Seisakusho, Japan; internal diameter, 0.5 mm) was inserted into the right ventricle via the right jugular vein to measure systolic pressure. Blood pressure was monitored using a physiological transducer (USB_DP_T, emka TECHNOLOGIES, France), amplifier system (USB_AMP_4BR, emka TECHNOLOGIES), and software system (iox2, emka TECHNOLOGIES). To evaluate right ventricular hypertrophy, Fulton's index was calculated as the ratio of right ventricle to left ventricle weight plus interventricular septum weight.

### 2.3 Sample collection

After catheterization, thoracotomy was performed and the lungs and heart were removed. Blood samples were taken at the same time. These organs were formalin-fixed and paraffin-embedded. Next, 5 μm-thick sections were cut using a microtome and placed onto adhesive glass slides (Cat#:CRE-01, Matsunami Glass, Japan). In addition, 8 μm-thick sections were collected onto laser-capture microdissection coupled with mass spectrometry (LCM)-compatible polyethylene terephthalate frame slides (Cat#: 50001–024, AMR, Japan).

### 2.4 Hematoxylin–eosin and toluidine blue staining

All sections were dewaxed and rehydrated by performing successive washes with xylene (2 × 30 s), 100% ethanol (2 × 30 s), 95% ethanol (1 × 30 s), 70% ethanol (1 × 30 s), and ultrapure water (1 × 30 s). Sections on adhesive glass slides were stained with hematoxylin and eosin for histological imaging. Sections on polyethylene terephthalate frame slides underwent toluidine blue staining for LCM, followed by immersion in 0.5% toluidine blue (Cat#36693, SERVA Electrophoresis, Germany) with ultrapure water for 20 s. Sections were washed five times in ultrapure water, immersed for 2 min in 95% ethanol, and immersed twice for 5 min in 100% ethanol. The sections were completely air-dried for at least 12 h prior to LCM.

### 2.5 Histological measurements

The histological measurement method was adopted from the procedure described by Shinohara et al. and the slides were analyzed in a blinded manner [12]. The external diameters of small PAs in the lung sections were measured along the shortest curvature. The percent medial wall thickness of the muscular arteries (outer diameter 50–200 μm) was calculated using the following formula: (external diameter–internal diameter)/external diameter × 100%. Complementary occluded lesions, onion skin lesions, and PLs were not included in the measurement of the percent medial thickness of the muscular arteries because actual wall thickening could not be measured. Images were acquired using a confocal microscopy (ix83; Olympus, Japan).

All small arteries (outer diameter < 50 μm) per lung section were assessed for occlusive lesions at 40× magnification. A vessel in which the lumen was partially (50%) or fully obstructed was defined as an occlusive lesion (Grade 2) [13]. Quantitative analysis was performed to determine the ratio of occlusive lesions among all the small PAs per lung section. The types of vessel occlusion were defined by the pattern of α-smooth muscle actin (αSMA)-positive cell distribution [14].

### 2.6 Laser-capture microdissection

**Fig. 1:**
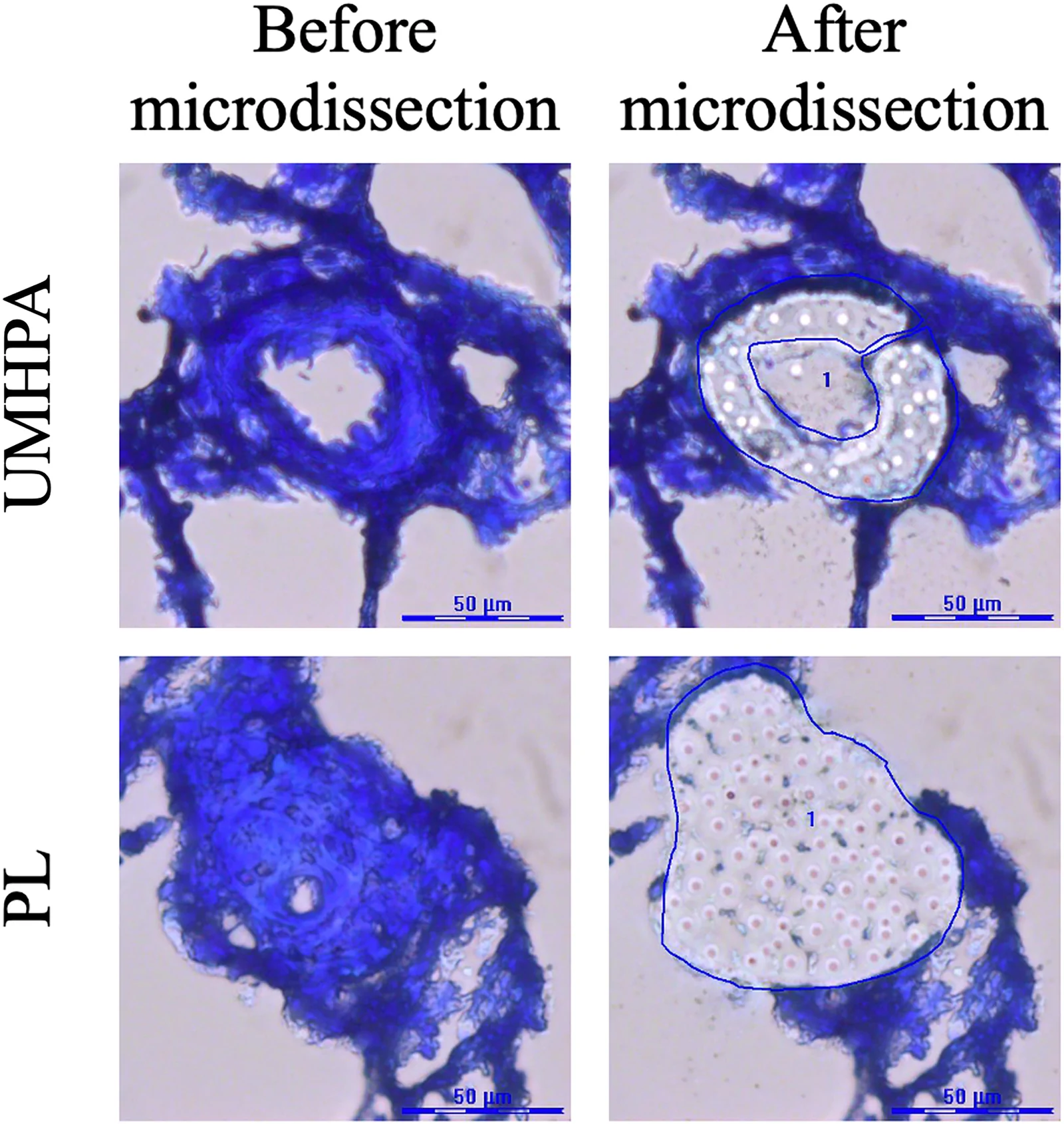
Study method. Rat tissues extraction by laser-capture microdissection for mass spectrometry (scale bar = 50 μm, toluidine blue stain). PL, plexiform lesion; UMHPA, unobstructed pulmonary artery with medial hypertrophy. (For interpretation of the references to colour in this figure legend, the reader is referred to the web version of this article.)

The slides used for sample collection were visually evaluated using toluidine blue staining, which has minimal impact on LC/MS. Furthermore, serial sections were stained with HE and α-SMA staining to confirm that the collected tissue was UMHPAs or PLs. Regions of UMHPAs and PLs were isolated using an LMD7000 system (Leica, Germany) at 20× magnification. Concentric laminar endometrial fibrosis (onion skin lesions) associated with PLs, was included in PLs. Each tissue was collected in a 2.5 mm^2^ area (*n* = 5 per group) as reported earlier [7], [8], [15]. Only UMHPAs from the 8w model and PLs from the 13w model were collected (Fig. 1).

UMHPAs and PLs were identified using toluidine blue staining. All regions isolated using LCM were collected into 50 μL ultrapure water in the caps of 0.5 mL collection tubes (Eppendorf, Germany). The time spent performing LCM depended on the type of region collected and ranged from 10 to 20 h. UMHPAs that required more time for collection in the required area were collected in two separate sessions. At the end of each LCM session, the collection tubes were centrifuged at 14,000 ×*g* for 2 min and stored at −80 °C.

### 2.7 Tissue digestion for mass spectrometry

This method was adapted from a modified version of the procedure described by Drummond et al. [15], [16]. First, tissue samples were thawed at room temperature (20–25 °C) and centrifuged at 14,000 ×*g* for 2 min followed by the addition of 50 μL 100 mM ammonium bicarbonate and 20% acetonitrile solution to the cap of the tube. The contents were pipetted up and down two or three times and centrifuged to collect the solution. If one sample was collected in two separate collection tubes, the solutions from the two tubes were mixed.

Next, the samples were incubated at 95 °C for 1 h, followed by incubation at 65 °C for 2 h to allow deparaffinization and reverse crosslinking of the proteins. At the end of the incubation, the samples were cooled to room temperature and centrifuged at 14,000 ×*g* for 3 min to collect the solution. The samples were then dried in a CentriVap concentrator (Labconco) and 90 μL 70% formic acid was added to the dried sample. The samples were then vortexed and incubated overnight at room temperature.

At the end of the incubation, the samples were sonicated for 5 min using a water bath sonicator (MCS-3, AS ONE, Japan) and vortexed three times. The samples were then completely dried using a CentriVap concentrator. The dried samples were resuspended into 150 μL 100 mM ammonium bicarbonate solution, pH 8.0. The samples were subsequently reduced with dithiothreitol (20 mM) at 57 °C for 1 h followed by alkylation with iodoacetamide (50 mM) at room temperature in the dark for 45 min. Next, sequence-grade modified trypsin (300 ng; Cat # V5280; Promega, USA) was added to each sample and samples were digested overnight on a shaker at room temperature.

The digestion was stopped by adding 0.5% trifluoroacetic acid. A 5 μL sample was used to quantify total peptide concentration using a quantitative fluorometric peptide assay kit (Cat# 23290, Thermo Fisher Scientific, USA). For all samples, the resulting peptides were desalted using Poros beads, as described previously [17]. In brief, a slurry of Poros R2 20 μm beads (Cat# 1112906, Thermo Fisher Scientific) was added to each sample to achieve a sample volume to Poros beads ratio of 9:1. The samples were shaken at 4 °C for 3 h and the beads were loaded onto equilibrated GL-Tips SDB (GL Sciences, Japan) using a microcentrifuge for 30 s at 4000 ×*g*. The Poros beads were further washed with 0.1% trifluoroacetic acid and then 0.5% acetic acid. Peptides were eluted by adding 40% acetonitrile in 0.5% acetic acid, followed by the addition of 80% acetonitrile in 0.5% acetic acid. Finally, the organic solvent was removed using a CentriVap concentrator.

### 2.8 Mass spectrometry data analysis

The peptides were analyzed via liquid chromatography tandem-mass spectrometry (LC–MS/MS) using an Orbitrap Fusion mass spectrometer (Thermo Fisher Scientific) coupled to an UltiMate3000 RSLCnano LC system (Dionex, Netherlands) and equipped with a nano high-performance liquid chromatography (HPLC) capillary column, 150 mm × 75 μm i.d. (Nikkyo Technos, Japan) and a nanoelectrospray ion source. Reversed-phase liquid chromatography was performed with a linear gradient (0 min, 5% B; 100 min, 40% B) of solvent A (2% acetonitrile with 0.1% formic acid) and solvent B (95% acetonitrile with 0.1% formic acid) at an estimated flow rate of 300 nL/min.

A precursor ion scan was performed using a mass-to-charge ratio (*m*/*z*) of 400–1600 prior to the MS/MS analysis. Tandem MS was performed by isolation width of 1.6 *m*/*z* with the quadrupole, HCD fragmentation with normalized collision energy of 35%, and rapid scan MS analysis of the ion trap. Only precursors with charge states of 2–6 were sampled for MS2. The dynamic exclusion duration was set to 15 s, with a tolerance of 10 ppm. The instrument was operated in top-speed mode with 3 s cycles.

Raw data were processed using Proteome Discoverer 1.4 (Thermo Fisher Scientific) in conjunction with the MASCOT search engine, version 2.7.0 (Matrix Science, USA) for protein identification. Peptides and proteins were identified against the rat protein database in UniProt (release 2021_02) with a precursor mass tolerance of 10 ppm and a fragment ion mass tolerance of 0.8 Da. The fixed modification was set to the carbamidomethylation of cysteine and variable modifications were set for the oxidation of methionine. Two missed cleavages were performed with trypsin.

The area of the extracted ion chromatogram at the MS/MS level was used for quantification. Only proteins with at least four valid values per group were used in the analysis. To those proteins, a constant-value imputation approach was applied as follows: for protein levels below the limit of quantification (LOQ), a value of LOQ × 0.5 was imputed using the LOQ of the respective protein. The fold-change (FC) was calculated using the mean of each group. Proteins upregulated in each group were identified using Student's *t*-test. Statistical significance was set at –Log_10_ (*P*) > 1.3 (*P* < 0.05). Proteins with absolute |Log_2_ FC| of at least 0.5 (FC **≈** 1.4) were considered differentially expressed proteins. Proteins detected in four or five samples in one group but not detected in the other group were also considered differentially expressed proteins. The amount of glyceraldehyde-3-phosphate dehydrogenase (−Log_10_ (*P*) = 0.15, |Log_2_ FC| = 0.1) in each sample was comparable, and the amount of protein in each sample was equal. The differentially expressed proteins obtained were searched in the literature for their involvement in PA remodeling in PAH.

### 2.9 Immunohistochemistry

All protocols followed the manufacturer's recommendations. The localization and intensity of immunoreactivity were determined by an investigator blinded to the experimental and control groups. The specificity of each antibody used for immunohistochemical staining was validated by the use of positive and negative controls. Primary antibodies specific to the following proteins were used: actin alpha cardiac muscle 1 (Gene Tex, Cat# GTX101876; 1:100 dilution), aldose reductase (Gene Tex, Cat# GTX113381; 1:100 dilution), αSMA (Sigma-Aldrich, Cat# C6198, RRID:AB_476856; 1:500 dilution), annexin A3 (Abcam, Cat# ab127924, RRID:AB_11143246; 1:500 dilution), calmodulin 1 (Affinity Biosciences, Cat# AF6353; 1:100 dilution), β-actin (Santa Cruz Biotechnology, Cat# sc-47,778, RRID:AB_626632ACTB; 1:500 dilution), calmodulin-like protein 3 (Proteintech, Cat# 17275–1-AP, RRID:AB_2878374; 1:200 dilution), calponin 1 (Santa Cruz Biotechnology, Cat# sc-58,707, RRID:AB_781770; 1:200 dilution), calponin 3 (Gene Tex, Cat# GTX106174; 1:100 dilution), caveolin 1 (Abcam Cat# ab32577, RRID:AB_725987; 1:2000 dilution), complement C3 (Santa Cruz Biotechnology, Cat# sc-28,294, RRID:AB_627277; 1:100 dilution), desmin (Santa Cruz Biotechnology, Cat# sc-65,983, RRID:AB_1122192; 1:100 dilution), complement C4 (Abbexa, Cat# abx175977; 1:100 dilution), EH domain-containing 2 (Proteintech, Cat# 11440–1-AP, RRID:AB_2097325; 1:400 dilution), fatty acid-binding protein 5 (Santa Cruz Biotechnology, Cat# sc-365,236, RRID:AB_10846034; 1:50 dilution), hnRNPA1 (Santa Cruz Biotechnology, Cat# sc-32,301, RRID:AB_627729; 1:50 dilution), matrix Gla protein (Invitrogen, Cat# PA5–96323; 1:100 dilution), migration inhibitory factor (Proteintech, Cat# 20415–1-AP, RRID:AB_10694820; 1:100 dilution), myosin heavy chain 10 (Santa Cruz Biotechnology, Cat# sc-376,954, RRID:AB_2928059; 1:100 dilution), myosin light chain 9 (Santa Cruz Biotechnology, Cat# sc-28,329, RRID:AB_2282358; 1:100 dilution), nidogen-2 (Santa Cruz Biotechnology, Cat# sc-377,424, RRID:AB_2819357; 1:100 dilution), pyruvate kinase 2 (Proteintech, Cat# 15822–1-AP, RRID:AB_1851537; 1:100 dilution), peroxiredoxin 2 (Proteintech Cat# 10545–2-AP, RRID:AB_2168202; 1:1200 dilution), parathymosin (Sigma-Aldrich, Cat# HPA03186; 1:1000 dilution), sulfated glycoprotein-1 (Santa Cruz, Cat# sc-390,184; 1:100 dilution), transgelin (Santa Cruz Biotechnology, Cat# sc-53,932, RRID:AB_1129519; 1:50 dilution), thymosin beta 4 (Biorbyt, Cat# orb2811; 1:100 dilution), and von Willebrand factor (Invitrogen, Cat# PA5–104687; 1:100 dilution).

### 2.10 Pathway analysis

For each protein upregulated in unobstructed pulmonary arteries with medial hypertrophy (UMHPAs) and PLs, pathway and process enrichment analyses were performed using Metascape (https://metascape.org/gp/index.html). Only terms with *P* < 0.01, a minimum count of 3, and an enrichment factor (ratio between the observed counts and counts expected by chance) >1.5 were collated and grouped into clusters based on membership similarities.

### 2.11 Serum hnRNPA1 concentration

Among the DEPs, we focused on elevated hnRNPA1 and examined the role of PAH remodeling of PA. Before functional analysis, serum hnRNPA1 concentration was determined using a rat hnRNPA1 ELISA kit (Cat# abx353728).

### 2.12 Immunofluorescence to investigate hnRNPA1 localization

We performed immunofluorescence on PLs to validate the cell types exhibiting elevated levels of hnRNPA1. Sections on adhesive glass slides were heated in the oven at 60 °C for 30 min. Sections were dewaxed and rehydrated by performing successive washes with Hemo-De (3 × 5 min), 100% ethanol (2 × 5 min), 95% ethanol (1 × 5 min), 70% ethanol (1 × 5 min), and PBS (3 × 5 min). Sections were heated in boiling 0.01 M citric acid (pH 6.0) for 10 min, allowed to cool (30 min), and washed with PBS for 3 × 5 min. Sections were treated with blocking solution (4% normal donkey serum and 0.01% Triton with PBS) for 30 min. Sections were incubated with primary antibodies in blocking solution overnight at 4 °C. Primary antibodies against the following proteins were used: αSMA (Proteintech Cat# 23081–1-AP, RRID:AB_2815024; 1:200 dilution), hnRNPA1 (Santa Cruz Biotechnology Cat# sc-32,301, RRID:AB_627729; 1:50 dilution), leukocyte common antigen (Abcam Cat# 20103–1-AP, RRID: AB_2716813; 1:1000 dilution) and platelet endothelial cell adhesion molecule (PECAM-1; Proteintech Cat# ab182981, RRID:AB_2920881; 1:2000 dilution). Sections were washed with PBS for 3 × 5 min. Sections were incubated with secondary antibodies in blocking solution for 60 min at room temperature in the dark. The following secondary antibodies were used: donkey anti-mouse IgG (H + L) highly cross-adsorbed secondary antibody, Alexa Fluor™ 555 (Invitrogen, Cat# A31570; 1:500 dilution) and donkey anti-rabbit IgG (H + L) highly cross-adsorbed secondary antibody, Alexa Fluor™ 488 (Invitrogen, Cat# A21206; 1:500 dilution). Sections were washed with PBS (3 × 5 min). Sections were mounted using ProLong™ Gold Antifade Mountant with 4′,6-diamidino-2-phenylindole (Cat# P36935, Thermo Fisher Scientific). The localization and intensity of imunoreactivity were determined by a blinded investigator.

### 2.13 Cell culture and characterization

Cell culture and characterization were performed using the supplemental methods. Control rat PASMCs (rPASMCs) were purchased from Cell Applications, USA (Cat# CAR35205a) and cultured in Dulbecco's Modified Eagle Medium (DMEM) containing 10% fetal bovine serum and 1% penicillin–streptomycin in a humidified incubator at 37 °C with 5% CO_2_. Passages 5–8 were used for all experiments. The cells were exposed to hypoxic conditions (5% O_2_) to simulate the effects of PAH [18]. Hypoxia was induced in an incubator (Cat# MCO-5MUV-PJ, PHC Holdings, Japan) by introducing nitrogen gas, and the oxygen concentration was monitored continuously using an oxygen sensor.

### 2.14 Small interfering RNA transfection

rPASMCs were pre-seeded at 1 × 10^4^ cells per well in a 96-well plate for the evaluation of cell proliferation or at 5 × 10^4^ cells per well in a 24-well plate for immunofluorescence and western blotting. Quiescence was induced by subjecting cells to serum starvation for 24 h and the cells were then transfected with hnRNPA1 siRNA (sense strand: GGA UGG AAG AGU UGU GGA A; antisense strand: UUC CAC AAC UCU UCC AUC C, Bioneer, Korea) or negative control siRNA (Cat # SN-1001, Bioneer) according to the manufacturer's instructions. Briefly, siRNA stock solution (10 μM) was diluted with minimum essential medium (MEM) (Opti-MEM, Cat# 31985062, Gibco, USA) and mixed with lipofectamine RNAiMAX (Cat# 13778100, Thermo Fisher Scientific) diluted in Opti-MEM and added to the cells. Subsequently, the transfected rPASMCs were cultured under normoxic or hypoxic conditions (5% O_2_) and their proliferation was evaluated at 0, 24, 48, and 72 h after siRNA transfection. Western blotting and immunofluorescence were performed after 72 h treatment.

### 2.15 Cell proliferation assay

Cell proliferation was measured using a cell counting kit-8 (CCK-8) assay (Cat#: CK04–01, Dojindo, Japan). The CCK-8 reagent was diluted in the culture medium at a ratio of 1:10 and incubated with rPASMCs for 2 h before the absorbance at 450 nm was measured using a microplate reader (Cytation, BioTek, USA). We also counted the number of rPASMCs adhering to the bottom of the 96-well plates 72 h after treatment using ImageJ (https://imagej.net/software/fiji/downloads) to verify the CCK-8 results. rPASMCs attached to the sidewalls of 96-well plates were not counted. Each experiment was performed independently six times (*n* = 6 per group).

### 2.16 Immunofluorescence of cultured rat PASMCs

The proliferative capacity of siRNA-transfected rat PASMC (rPSAMC) was evaluated using fluorescent staining. After 72 h incubation, siRNA-transfected rPASMCs were fixed in 4% paraformaldehyde, washed with PBS, and incubated with a blocking solution (5% normal goat serum and 0.3% Triton X-100 in PBS), Hoechst dye, and antibodies according to the manufacturer's instructions. To assess cell proliferation and apoptosis, rPASMCs were stained with Ki-67 (Cell Signaling Technology, Cat# 11882, RRID:AB_2687824) and cleaved caspase-3 (Cell Signaling Technology, Cat# 9669, RRID:AB_2069869) specific antibodies conjugated with Alexa Fluor 488. Each experiment was performed independently six times (*n* = 6 per group).

### 2.17 Protein extraction from cultured rPASMCs

Proteins were extracted from rPASMCs using the Minute Total Protein Extraction Kit for Animal Cultured Cells and Tissues (Cat# SD-001, Invent Biotechnologies, USA), according to the manufacturer's instructions.

### 2.18 Sample preparation for automatic capillary western blotting

Western blotting was performed using an automatic capillary western blotting device (Simple Western; Jess, ProteinSimple, USA). For detecting β-actin, hnRNPA1, IQ motif containing GTPase activating protein 1 (IQGAP1), and pyruvate kinase M 2 (PKM2), a Jess/Wes 12 to 230 kDa separation module, 8 × 25 capillary cartridges, and an anti-rabbit/goat/mouse detection module kit were used. Sample loading and reagent preparation were performed according to manufacturer's instructions. Primary antibodies against the following proteins were used: β-actin (Santa Cruz Biotechnology, Cat# sc-47,778, RRID:AB_626632; 1:200 dilution), hnRNPA1 (Santa Cruz Biotechnology Cat# sc-32,301, RRID:AB_627729; 1:10 dilution), IQGAP1 (Santa Cruz Biotechnology, Cat# sc-376,021, RRID:AB_10988556; 1:400 dilution) and PKM2 (Proteintech Cat# 15822–1-AP, RRID:AB_1851537; 1:50 dilution). Chemiluminescent signals were detected and quantified automatically using Simple Western Jess, and Compass software 6.1.0 (ProteinSimple) was used to process and analyze the data. Protein expression was quantified by measuring the optical density of the area of their corresponding bands on the blots. To normalize expression in the same sample, the housekeeping protein β-actin was used as the reference. Each experiment was performed independently six times (*n* = 6 per group).

### 2.19 Statistical analysis

Statistical analyses are detailed in the Supplemental Methods. JMP 13 Pro software (SAS Institute, Cary, NC, USA) was used for all analyses. Data are presented as the mean ± SE for continuous variables. One-way analysis of variance was used to compare variables among three or more groups, followed by Tukey's multiple comparison test. Results with *P* < 0.05 were considered statistically significant.

## 3 Results

### 3.1 Hemodynamics and histopathology of SuHx rats

**Fig. 2:**
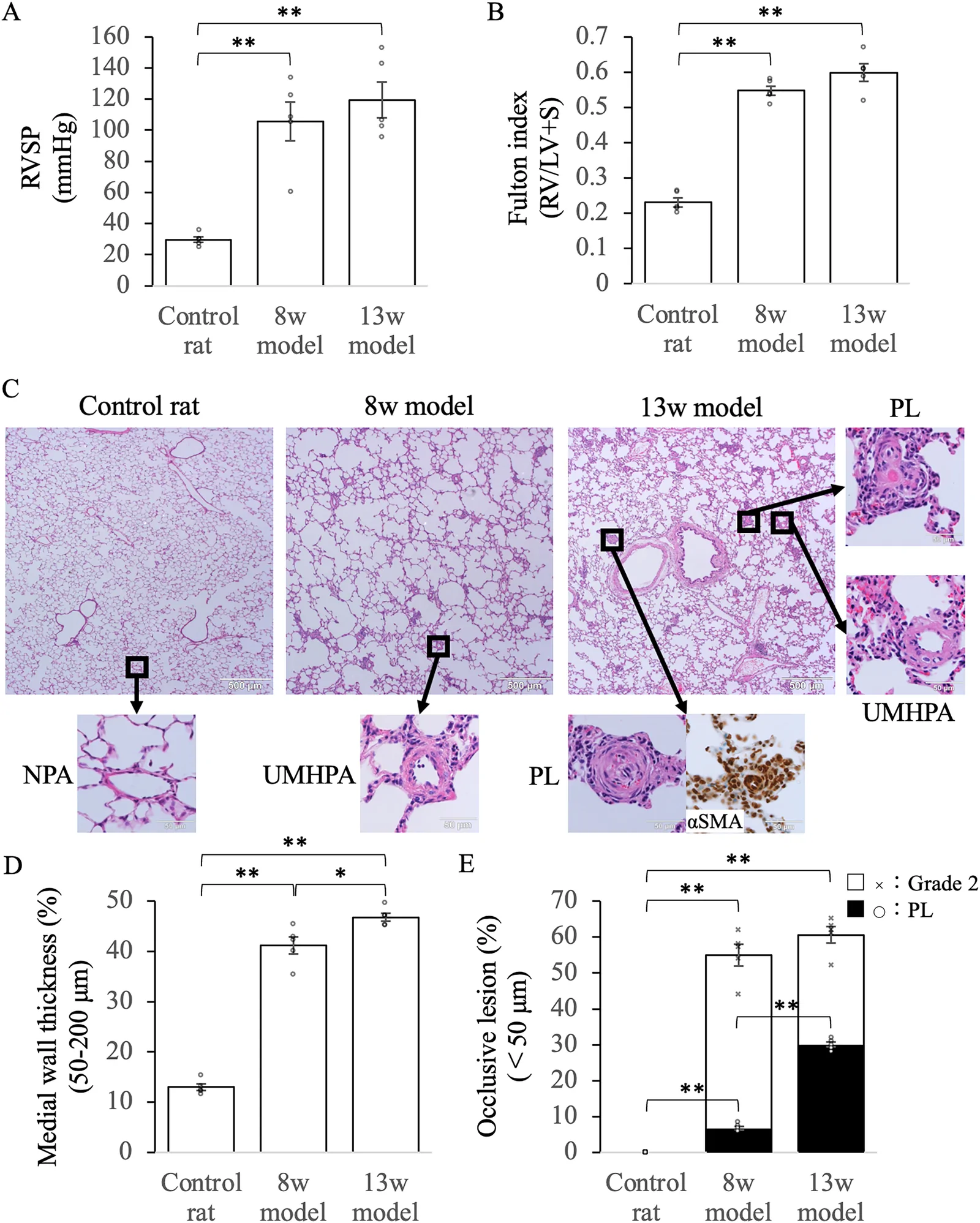
PAH progression in the SuHx rat model. (A) Right ventricular systolic pressure (RVSP). (**B**) Fulton's index, weight ratio of the right ventricle to the left ventricle + septum (RV/LV + S). (**C**) Hematoxylin–eosin staining showing the pathological progression in SuHx rats (scale bar = 500 μm; inset, scale bar = 50 μm). (**D**) Percentage of medial wall thickness. (**E**) Percentage of occlusive lesions among the vessels. *P < 0.05; **P < 0.01 (one-way ANOVA followed by Tukey's multiple-comparison test). Values are the mean ± SE, *n* = 5 per group. αSMA = α-smooth muscle actin; PL = plexiform lesion; SuHx = SU5416 combined with hypoxia; 8w = 8-week; 13w = 13-week; NPA = normal pulmonary artery; PL = plexiform lesion; UMHPA = unobstructed pulmonary artery with medial hypertrophy.

Relative to the level in control rats (29.6 ± 1.9 mmHg), right ventricular systolic pressure (RVSP) increased in rats in both 8-week (8w; 105.6 ± 12.5 mmHg, *P* < 0.01) and 13-week (13w; 119.4 ± 11.5 mmHg, *P <* 0.01) models (Fig. 2A). Relative to the level in control rats (0.23 ± 0.01), Fulton's index increased in rats in both 8w (0.55 ± 0.01, *P* < 0.01) and 13w (0.60 ± 0.02, *P <* 0.01) models (Fig. 2B).

Fig. 2C to 2E shows the pathological progression in SuHx rats. The medial wall thickness of the control rats was thin (13.0 ± 0.7%), and no PLs (0.0 ± 0.0%) were observed. In the 8w model, few PLs (6.8 ± 0.6%) were noted, along with several UMHPAs with thickened medial wall thickness (41.2 ± 1.7%, *P* < 0.01), although not completely occluded; however, in the 13w model, many PLs (30.0 ± 0.7%, *P* < 0.01) were present.

### 3.2 LCM–MS

We attempted to extract normal PA tissue from control rats but could not obtain sufficient amounts for LCM–MS. Therefore, PLs in the 13w model were compared with UMHPAs during remodeling in the 8w model (Fig. 1). LCM–MS helped identify 718 proteins, with 58 DEPs among these regions. Of these, 31 DEPs were upregulated in UMHPAs during remodeling, and 27 DEPs were upregulated in PLs. Alpha-cardiac muscle 1 was the most upregulated protein in UMHPAs, followed by transgelin, histone H2A.Z, and annexin A3 (Table S1). In PLs, thymosin β4 was the most upregulated protein, followed by matrix Gla protein, CALML3, and C4 (Table S2).

### 3.3 Immunohistochemistry

**Fig. 3:**
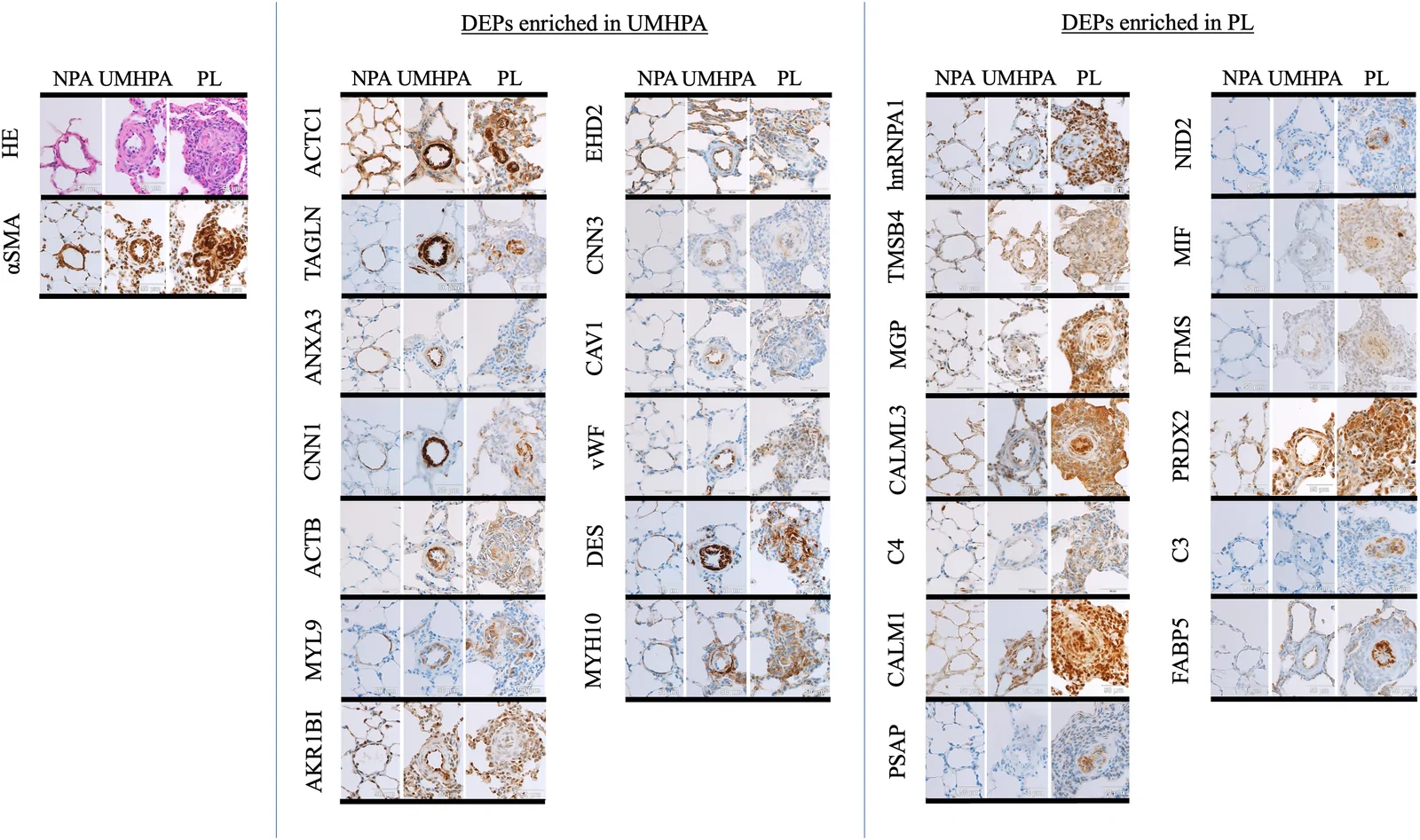
DEPs in normal PAs, UMHPAs, and PLs. Hematoxylin–eosin staining results of normal PAs, UMHPAs, and PLs and immunohistochemistry results of αSMA and each DEP are shown (scale bar = 50 μm). ACTB = β-actin; ACTC1 = actin alpha-cardiac muscle 1; AKR1B1 = aldo-keto reductase family 1 member B; αSMA = α-smooth muscle actin; ANXA3 = annexin A3; CALM1 = calmodulin 1; CALML3 = calmodulin-like 3; CAV1 = caveolin 1; CNN1 = calponin 1; CNN3 = calponin 3; C3 = complement C3; C4 = complement C4; DEP = differentially expressed protein; DES = desmin; EHD2 = EH domain-containing 2; FABP5 = fatty acid-binding protein 5; HE = hematoxylin–eosin; hnRNPA1 = heterogeneous nuclear ribonucleoprotein A1; MGP = matrix Gla protein**;** MIF = macrophage migration inhibitory factor; MYH10 = myosin heavy chain 10; MYL9 = myosin light chain 9; NID2 = nidogen 2; NPA = normal pulmonary artery; PL = plexiform lesion; PRDX2 = peroxiredoxin 2; PSAP = prosaposin; PTMS = parathymosin; TAGLN = transgelin; TMSB4 = thymosin β4; UMHPA = unobstructed pulmonary artery with medial hypertrophy; vWF = von Willebrand factor.

To validate the proteome results and explore the cellular location of DEPs, we performed immunostaining of the top DEPs that were altered according to LCM–MS and visually confirmed the predominant changes (Fig. 3). The top DEPs were less expressed in normal PAs compared with those in UMHPAs and PLs. Many of the upregulated DEPs in UMHPAs were proteins expressed on cells lining the lumen of PAs. Upregulated DEPs, including hnRNPA1, were highly expressed in PLs compared with those in normal PAs and UMHPAs.

### 3.4 Pathway analysis

**Fig. 4:**
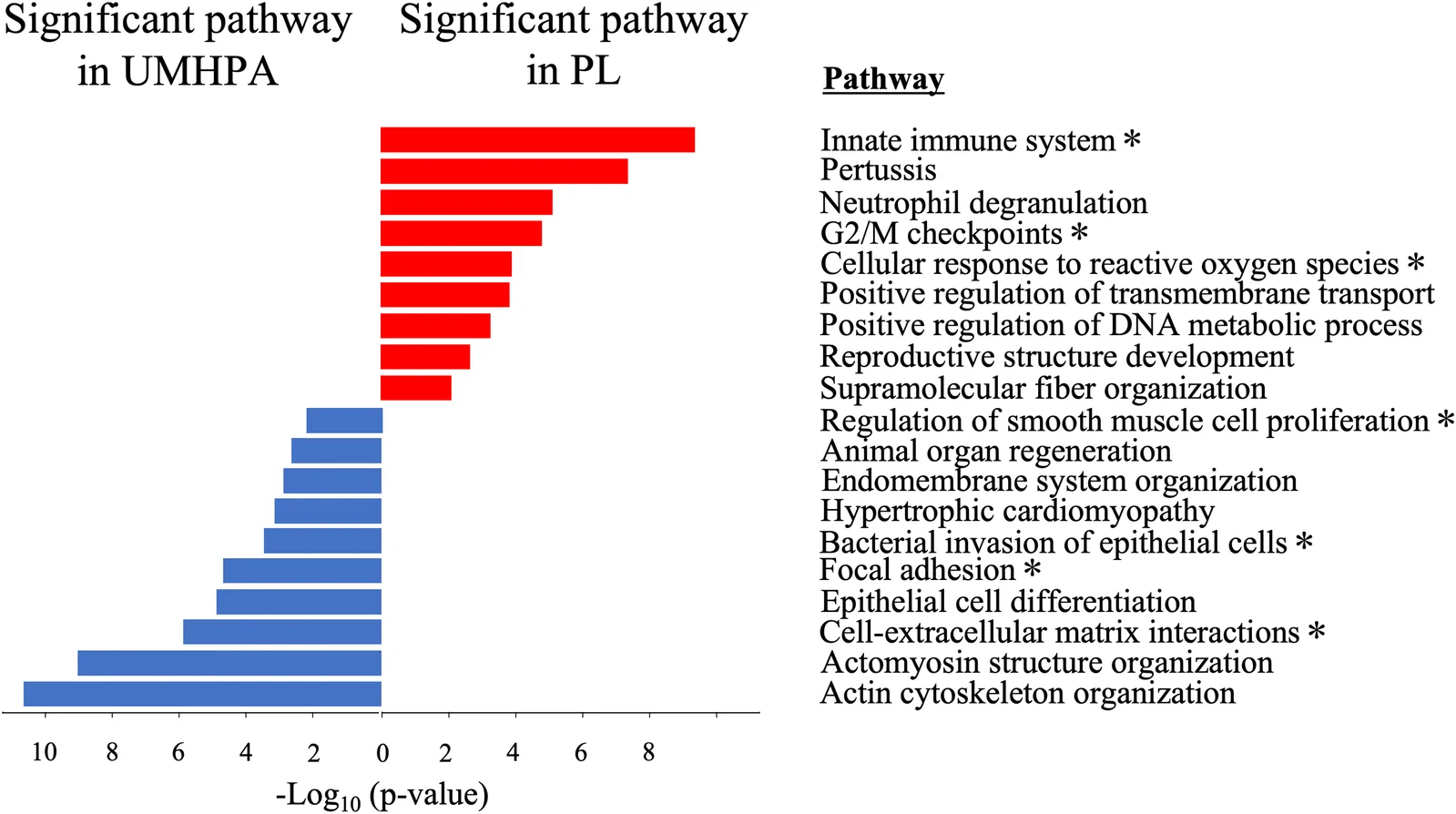
Significant pathways in UMHPAs and PLs. Red bars indicate significant pathways in PLs. Blue bars indicate significant pathways in UMHPAs. Asterisks indicate pathways reported to be related to pulmonary arterial hypertension. PL = plexiform lesion; UMHPA = unobstructed pulmonary artery with medial hypertrophy. (For interpretation of the references to colour in this figure legend, the reader is referred to the web version of this article.)

DEPs in UMHPAs and PLs were subjected to pathway and process enrichment analyses (Fig. 4). The top three protein clusters upregulated in UMHPAs consisted of actin cytoskeleton organization (GO:0030036), focal adhesion (rno04510), and cell–extracellular matrix interactions (R-RNO-446353), whereas those in PLs consisted of innate immune system (R-RNO-168249), pertussis (rno05133), and G2/M checkpoints (R-RNO-69481) (Fig. 4).

### 3.5 Candidate protein involved in PA remodeling in PAH and PLs

From a literature search, we determined that hnRNPA1, which shows elevated levels in PLs, might be a candidate protein involved in PA remodeling in PAH and PLs, given that it is an RNA-binding protein (RBP) and considering the association of RBPs with PA remodeling in PAH [19]. Therefore, we performed a functional analysis of hnRNPA1.

The variability of serum hnRNPA1 in SuHx rats was assessed before functional analysis of hnRNPA1. Relative to the level in control rats (23.7 ± 2.3 ng mL^−1^), serum hnRNPA1 concentration increased in the 8w (70.7 ± 12.2 ng mL^−1^, *P* < 0.01) and 13w (63.1 ± 14.3 ng mL^−1^, *P <* 0.01) models (Fig. S1). This indicates that hnRNPA1 levels are significantly elevated both in the PAH lesions and sera of SuHx rats.

### 3.6 Investigation of localization of hnRNPA1 via immunofluorescence

**Fig. 5:**
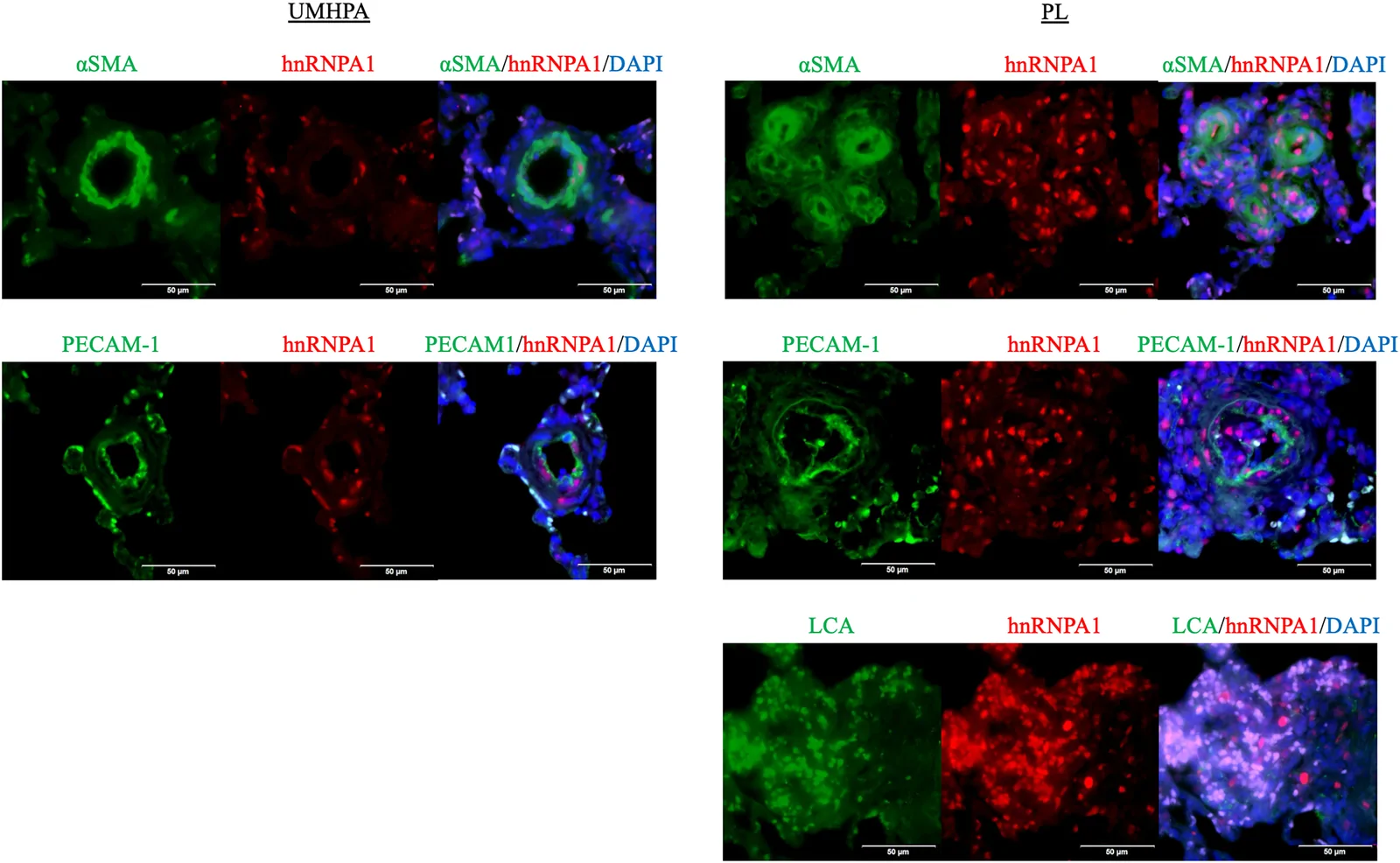
Localization of hnRNPA1 in UMHPAs and PLs. Staining for hnRNPA1, LCA, and PECAM-1 with DAPI in UMHPAs and PLs (scale bar = 50 μm). αSMA = α-smooth muscle actin; DAPI = 4′,6-diamidino-2-phenylindole; hnRNPA1 = heterogeneous nuclear ribonucleoprotein A1; LCA = leukocyte common antigen; PECAM-1 = platelet and endothelial cell adhesion molecule 1; PL = plexiform lesion; UMHPA, unobstructed pulmonary artery with medial hypertrophy.

Fig. 5 shows the localization of hnRNPA1. Compared to UMHPAs, immunofluorescence also clearly showed enhanced hnRNPA1 expression in PLs. In UMHPAs, some α-smooth muscle actin (αSMA)-positive cells (derived from PASMC-derived cells) were hnRNPA1-positive, but PECAM-1-positive cells (pulmonary artery endothelial cell-derived cells) were hnRNPA1-negative. In PLs, αSMA-positive cells and leukocyte common antigen-positive cells (immune cell-derived cells) showed strong positivity for hnRNPA1, whereas PECAM-1-positive cells were hnRNPA1-negative. This finding indicates that hnRNPA1 expression is enhanced in the cellular composition of PLs, specifically in PASMC-derived cells and immune cell-derived cells.

### 3.7 siRNA inhibition of hnRNPA1 in normal mature and hypoxia-treated rPASMCs

**Fig. 6:**
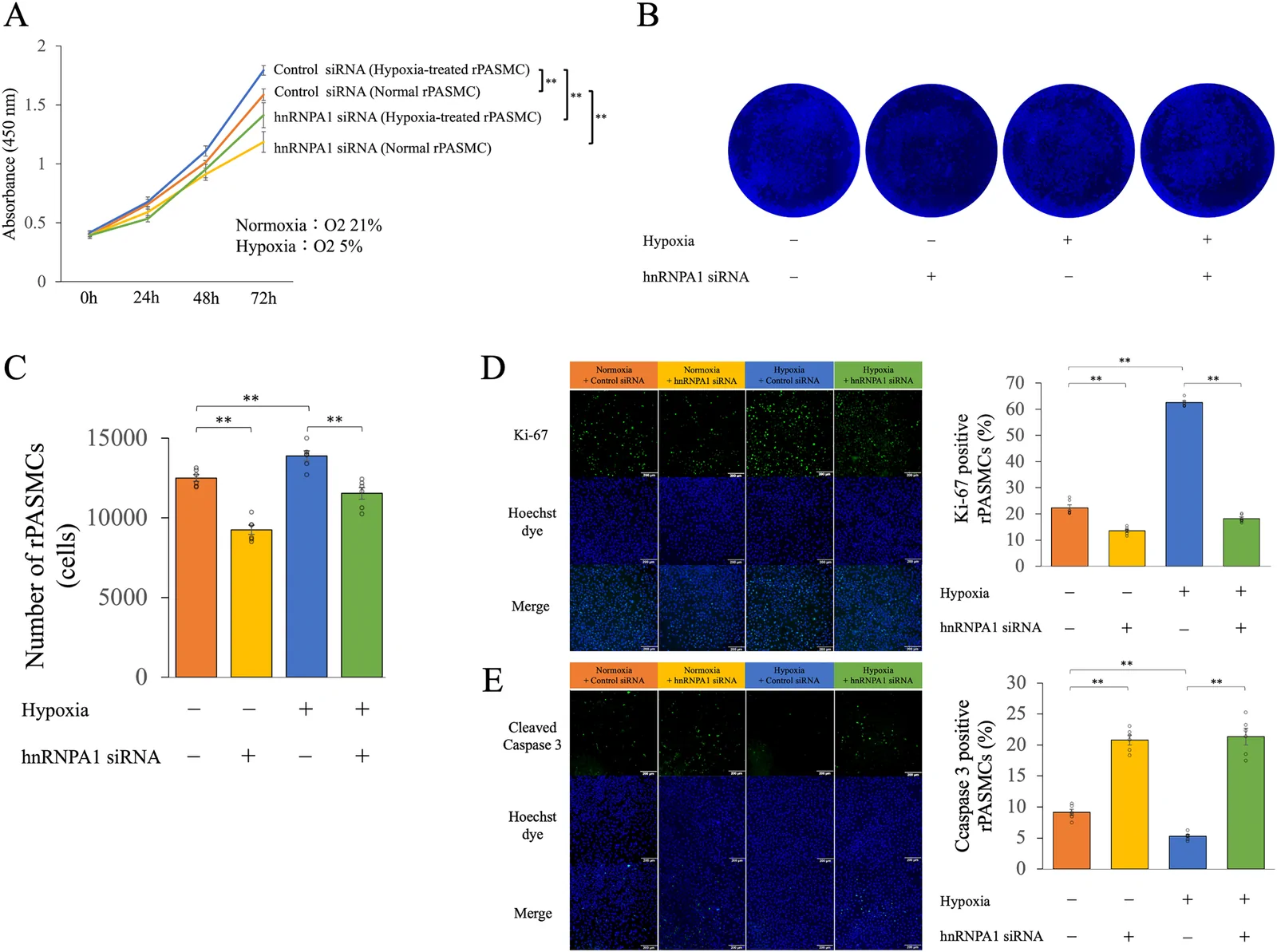
Effect of hnRNPA1 on proliferation and apoptosis in hypoxia-treated rPASMCs. (A) Proliferative capacity of hnRNPA1 siRNA- and control siRNA-treated rPASMCs (CCK-8 assay). **(B)** Staining with Hoechst dye 72 h after siRNA transfection. **(C)** Number of rPASMCs adhering to the bottom of 96-well plates 72 h after siRNA transfection. **(D)** Proliferative capacity of hnRNPA1 siRNA-and control siRNA-treated rPASMCs (Ki-67 staining). **(E**) Apoptosis in hnRNPA1 siRNA- and control siRNA-treated rPASMCs (staining for cleaved caspase 3). Orange, yellow, blue, and green colors show normoxia + control siRNA, normoxia + hnRNPA1 siRNA, hypoxia + control siRNA, and hypoxia + hnRNPA1 siRNA, respectively. *P < 0.05; **P < 0.01 (one-way ANOVA followed by Tukey's multiple-comparison test). Values are the mean ± SE, *n* = 6 per group. CCK = cell counting kit; hnRNPA1 = heterogeneous nuclear ribonucleoprotein A1; rPASMC = rat pulmonary artery smooth muscle cell; siRNA = small interfering RNA. (For interpretation of the references to colour in this figure legend, the reader is referred to the web version of this article.)

To analyze the function of hnRNPA1, which may be involved in PA remodeling in PAH and PLs, rPASMC knockdown experiments were performed using siRNAs because αSMA-positive cells were hnRNPA1-positive following immunofluorescence. Fig. 6A illustrates the proliferative capacity of rPASMCs (measured using the CCK-8 assay). Fig. 6B shows rPSAMCs stained with Hoechst dye 72 h after siRNA transfection, whereas Fig. 6C shows the number of rPASMCs adhering to the bottom of 96-well plates 72 h after siRNA transfection. Fig. 6D and E show the proliferative capacity (assessed by Ki-67-positive cell content) and apoptosis (assessed by cleaved caspase-3-positive cell content) of rPASMCs, respectively, demonstrating that transfection of normal rPASMCs with hnRNPA1 siRNA reduced their proliferative capacity and increased apoptosis. We used hypoxia-treated rPASMCs to simulate arterial muscle cells in pulmonary vascular lesions. Hypoxia-treated rPASMCs exhibited higher proliferative capacity and suppressed apoptosis compared with normal rPASMCs, whereas transfection with hnRNPA1 siRNA strongly suppressed proliferative capacity and increased apoptosis.

**Fig. 7:**
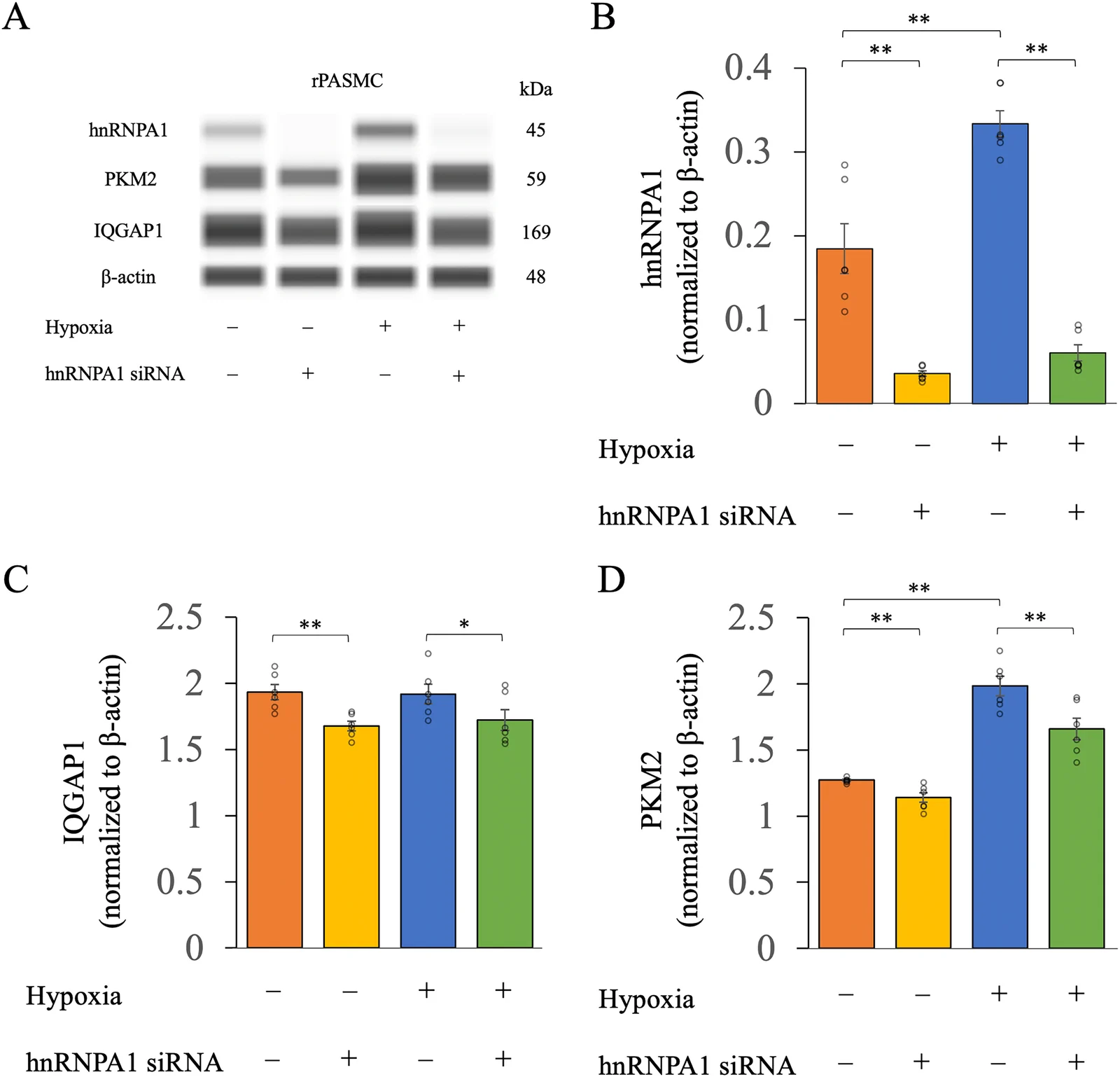
Effect of hnRNPA1 siRNA on protein expression in hypoxia-treated rPASMCs. (A), hnRNPA1, IQGAP1, PKM2 and β-actin protein expression in normal rPASMCs and hypoxia-treated rPASMCs treated with negative control siRNA or hnRNPA1 siRNA. **(B—D)**, Ratio of hnRNPA1/β-actin **(B)** PKM2/β-actin **(C)** and IQGAP1/β-actin **(D)** in normal rPASMCs and hypoxia-treated rPASMCs treated with negative control or hnRNPA1 siRNAs. Orange, yellow, blue, and green colors show normoxia + control siRNA, normoxia + hnRNPA1 siRNA, hypoxia + control siRNA, and hypoxia + hnRNPA1 siRNA, respectively. *P < 0.05; **P < 0.01 (one-way ANOVA followed by Tukey's multiple-comparison test). Values are the mean ± SE, *n* = 6 per group. hnRNPA1 = heterogeneous nuclear ribonucleoprotein A1; IQGAP1 = IQ motif containing GTPase activating protein 1; PKM2 = pyruvate kinase M 2; rPASMC = rat pulmonary artery smooth muscle cell; siRNA = small interfering RNA. (For interpretation of the references to colour in this figure legend, the reader is referred to the web version of this article.)

Automatic capillary western blotting analysis using Simple Western (Fig. 7A to 7D) showed that in normal rPASMCs, hnRNPA1 was firmly suppressed by hnRNPA1 siRNA; the expression of the scaffold protein IQGAP1, which is reportedly associated with hnRNPA1 [20], was slightly reduced; and PKM2 expression, which is reportedly involved in cell proliferation, was also reduced. The higher proliferative capacity of hypoxia-treated rPASMCs than that of normal rPASMCs could be associated with elevated hnRNPA1 and PKM2 expression. Knockdown of hnRNAP1 in hypoxia-treated rPASMCs also resulted in markedly decreased hnRNPA1 and PKM2 expression and cell proliferation.

## 4 Discussion

DEPs involved in PA remodeling in PAH and PLs were explored via LCM–MS. To our knowledge, our study is the first to directly examine DEPs in unobstructed peripheral PAs and PLs in this way. hnRNPA1 was among the DEPs and hnRNPA1 expression is enhanced in the cellular composition of PLs, specifically in PASMC-derived cells and immune cell-derived cells. Finally, hnRNPA1 was shown to be expressed in rPASMCs as well as being involved in their proliferation.

In previous studies, LCM–MS was applied to fresh [21], flash-frozen [22], and FFPE tissues [7], [8]. We first conducted the experiment using frozen sections but had difficulty in visually identifying the small UMHPAs and obtaining the necessary amount of protein for analysis. Although the protein detection rate is higher when fresh or flash-frozen tissues are used instead of FFPE tissues [21], [22], FFPE sections were selected for use because the luminal structure is maintained, which can be easily viewed in the sections. Despite the disadvantage of lower protein detection, we detected protein amounts similar to those previously reported for FFPE tissues [7], [8], [15], [16] and, following previously published methods, we were able to effectively analyze the FFPE tissue using LCM–MS.

A few LCM–MS studies have previously targeted pulmonary vessels; however, this is the first report examining PLs [7], [8]. A literature search was conducted to understand the functions of the DEPs detected by our proteomic analysis. Many retrieved proteins are known and reportedly associated with PA remodeling in PAH. In UMHPAs, levels of annexin A3, CAV1, myeloid-associated differentiation marker, transgelin, and transforming growth factor beta-1-induced transcript 1 protein, which are shown to be associated with PA remodeling in PAH [9], [23], [24], [25], [26], [27], were elevated. In PLs, in addition to elevated levels of complement C3, fatty acid-binding protein 5, and macrophage migration inhibitory factor, which are reportedly associated with PA remodeling in PAH, and levels of thymosin β4 and peroxiredoxin 2, which are reportedly protective against PA remodeling in PAH, were elevated [28], [29], [30], [31]. Thus, even in PAH lesions, elevated levels of proteins varied according to lesion status. Moreover, we identified several DEPs, including hnRNPA1; however, their roles in PA remodeling in PAH have yet to be established. A similar approach has been reported using transcriptome analysis to compare gene expression in PLs and UMHPAs, in which complement C3, calponin 1, transgelin, thymosin β4, matrix Gla protein, myosin heavy chain 11 and MYL9 exhibited similar behavior to that observed in our study, but not all DEPs were matched [10]. Immunostaining for several DEPs were consistent with those obtained from our proteome analysis. The differences between the results of proteomic analysis and transcriptome analysis can be attributed to species differences and the inherent discrepancy between gene and protein expression levels, which may not always align perfectly [32]. Furthermore, DEPs that could not be detected through proteomic analysis of the whole lung in the PAH models were detected using laser microdissection to extract only the lesions [33].

Among the pathways enhanced in UMHPAs in the enrichment analyses, “cell–extracellular matrix interactions,” “focal adhesion,” “bacterial invasion of epithelial cells,” and “regulation of smooth muscle cell proliferation” are related to PA remodeling in PAH [34], [35], [36], [37]. Similarly, among the pathways enhanced in PLs, “innate immune system,” “G2/M checkpoints,” and “cellular response to reactive oxygen species” are related to PA remodeling in PAH [38], [39], [40]. Thus, enhancement of different pathways in UMHPAs and PLs reflect the involvement of DEPs in these pathological conditions. This indicates that therapeutic targets may differ depending on the disease status and severity.

Based on our results, we focused on hnRNPA1, an RBP that has recently attracted attention for its relationship with PA remodeling in PAH [41]. Polypyrimidine tract binding protein 1, hnRNPA2/B1, and nucleoli are RBPs strongly associated with PA remodeling in PAH [41], [42], [43], [44]. In mature vascular smooth muscle cells, hnRNPA1 siRNA transfection increases the expression of scaffold protein IQGAP1, enhances proliferative capacity, and enables atherosclerosis progress [20]. In contrast, hnRNPA1 promotes the proliferative capacity of PASMCs in patients with chronic thromboembolic pulmonary hypertension and its level is increased in patients with hypoxia and PAH [41], [45]. Thus, hnRNPA1 exhibits dual effects, and its involvement in PA remodeling in PAH remains to be elucidated. The role of hnRNPA1 is also of interest in cancer growth, as it reportedly enhances the proliferation and migratory ability of undifferentiated cells by increasing PKM2 expression because hnRNPA1 can facilitate the switch from PKM1 to PKM2 [46]. Based on these contradictory results, we hypothesized that hnRNPA1 inhibits the proliferative capacity of mature differentiated PASMCs but enhances the proliferative capacity of cancer-like undifferentiated PASMCs.

Before performing functional analysis of hnRNPA1 on rPASMCs, we confirmed whether PASMC-derived cells in PLs enhanced hnRNPA1 expression. We confirmed that hnRNPA1 expression was enhanced in the cellular composition of PLs, specifically in PASMC-derived cells and immune cell-derived cells. Next, we performed knockdown experiments in normal and hypoxia-treated rPASMCs. Following siRNA transfection of normal rPASMCs, the expression of IQGAP1 and PKM2 as well as the proliferative capacity were reduced. Hypoxia-treated rPASMCs exhibited similar features. Therefore, siRNA transfection reduced the proliferative capacity in rPASMC under both conditions, indicating that their kinetics were different from those of systemic VSMCs, similar to a previous report on chronic thromboembolic pulmonary hypertension [45]. Our findings contribute to our understanding of PAH because, although the diseases are similar in some ways, PAH and chronic thromboembolic pulmonary hypertension have completely different pathological conditions. Suppressing hnRNPA1 in the early stages of PAH may be associated with a low risk of worsening PA remodeling in PAH, although the risk of atherosclerosis in the systemic vessels should be considered. Results of previous studies and the present study provide evidence that similar to other RBPs, suppressing the excessive increase in hnRNPA1 in undifferentiated PASMCs and immature PLs may suppress PA remodeling in PAH and PLs [41], [42], [43], [44].

The strengths of this study are as follows. For the first time, we comprehensively examined the direct proteomic remodeling in PLs by LCM–MS. We identified several DEPs, prior to and following PL formation, with unclear relevance to PA remodeling in PAH, which were not detected by previously reported transcriptome analysis and whole lung analysis. There were significant differences in protein and pathway enrichment between UMHPAs and PLs, which may lead to the identification of different therapeutic targets. In addition, hnRNPA1 is strongly expressed in PLs, and its suppression in normal and hypoxia-treated rPASMCs showed that hnRNPA1 might be strongly associated with PA remodeling in PAH and PLs. Thus, hnRNPA1 is a potentially useful target for the development of therapies for severe PAH.

This study has several limitations. First, normal PA tissue for LCM-MS analysis were not included as controls. Second, functional analyses of other DEPs are ongoing, and other important DEPs may also contribute to pulmonary vascular remodeling. Third, we used hypoxia-treated rPASMCs; therefore, the results may differ from those obtained using primary PASMCs isolated from rats with PAH. Fourth, while hnRNPA1 levels were significantly elevated in PASMC-derived cells in PLs, a similar increase was observed in immune cell-derived cells in PLs, suggesting that hnRNPA1 derived from these cells may enhance cell proliferation through paracrine mechanisms. Fifth, the functional role of hnRNPA1 in vivo was not directly evaluated in this study. Sixth, our functional analyses of hnRNPA1 were performed in rats, and the findings may not be directly applicable to humans. Therefore, human validation is required to determine the clinical relevance of hnRNPA1 in PAH. Although human samples are associated with various clinical variables, including disease stage and medication use, studies using a uniform animal model may facilitate the identification of candidate DEPs. Future studies should investigate the functional significance of hnRNPA1 using in vivo models and samples from patients with PAH.

## 5 Conclusions

We detected several differentially expressed proteins (DEPs) that may be associated with pulmonary vascular remodeling in PAH and PLs and may represent potential therapeutic targets. Our findings identify hnRNPA1 as a candidate associated with pulmonary vascular remodeling in PAH and PLs. Further studies are warranted to validate its biological significance and determine its potential utility as a therapeutic target or biomarker. Currently, few therapeutic options are available for pulmonary lesions (PLs) in patients with severe PAH. Our findings provide new insights into the mechanisms underlying pulmonary vascular remodeling in PAH and PLs and may contribute to the identification of potential therapeutic strategies for PLs.

## CRediT authorship contribution statement

**Yoshihito Morimoto:** Writing – review & editing, Writing – original draft, Visualization, Validation, Software, Resources, Methodology, Investigation, Funding acquisition, Formal analysis, Data curation, Conceptualization. **Yoshiaki Sato:** Writing – review & editing, Methodology. **Katsuhiro Kato:** Writing – review & editing, Methodology. **Hidenori Yamamoto:** Writing – review & editing, Methodology. **Kentaro Suzuki:** Writing – review & editing, Investigation. **Kiyotaka Go:** Writing – review & editing, Investigation. **Yoshie Fukasawa:** Writing – review & editing, Investigation. **Naoki Ohashi:** Writing – review & editing. **Yoshiyuki Takahashi:** Writing – review & editing. **Taichi Kato:** Writing – review & editing, Visualization, Validation, Supervision, Software, Resources, Project administration, Methodology, Investigation, Funding acquisition, Formal analysis, Data curation, Conceptualization.

## Funding

This work was partially supported by JSPS KAKENHI (Grant Numbers 20 K08155 and 23 K07329) and JST SPRING (Grant Number JPMJSP2125).

## Declaration of competing interest

The authors declare the following financial interests/personal relationships which may be considered as potential competing interests: Taichi Kato reports financial support was provided by JSPS KAKENHI. Yoshihito Morimoto reports financial support was provided by JST SPRING. If there are other authors, they declare that they have no known competing financial interests or personal relationships that could have appeared to influence the work reported in this paper.

## Data Availability

The data that support the findings of this study are available from the corresponding author upon reasonable request.
