# Supplementary material for: Heterogeneous nuclear ribonucleoprotein A1 as a candidate associated with pulmonary vascular remodeling in pulmonary arterial hypertension
Source: J Mol Cell Cardiol Plus. 2026 Aug 31;18:100867. doi: 10.1016/j.jmccpl.2026.100867 (PMC13571748; doi:10.1016/j.jmccpl.2026.100867)
Supplement: Supplementary file 1 [file mmc1.docx]

**Supplemental Information**

**Heterogeneous nuclear ribonucleoprotein A1 as a candidate associated with pulmonary vascular remodeling in pulmonary arterial hypertension**

Yoshihito Morimoto^a,b^, Yoshiaki Sato^c^, Katsuhiro Kato^d^, Hidenori Yamamoto^a^, Kentaro Suzuki^a^, Kiyotaka Go^a^, Yoshie Fukasawa^a^, Naoki Ohashi^a^, Yoshiyuki Takahashi^a^, Taichi Kato^a^

^a^Department of Pediatrics, Nagoya University Graduate School of Medicine, Nagoya, Aichi, Japan

^b^Department of Pediatric Cardiology, National Cerebral and Cardiovascular Center, Suita, Osaka, Japan

^c^Division of Neonatology, Center for Maternal-Neonatal Care, Nagoya University Hospital, Nagoya, Aichi, Japan

^d^Department of Cardiology, Nagoya University Graduate School of Medicine, Nagoya, Aichi, Japan

# Supplemental Figure

## Figure S1. Serum hnRNPA1 concentration in SuHx rats. Comparison of serum hnRNPA1 concentrations in control rats in the 8- and 13-week models. **P* < 0.05; ***P* < 0.01 (one-way ANOVA followed by Tukey’s multiple-comparison test). Values are the mean ± SE, n = 5 per group.

## hnRNPA1 = heterogeneous nuclear ribonucleoprotein A1; SuHx = SU5416 combined with hypoxia.

## Figure S2. The full uncropped Blots image of Figure 7A.

## hnRNPA1 = heterogeneous nuclear ribonucleoprotein A1; IQGAP1 = IQ motif containing GTPase activating protein 1; PKM2 = pyruvate kinase M 2; siRNA = small interfering RNA.

## Supplemental Tables

## Table S1. Upregulated proteins in UMHPAs.

| **Protein ID** | **Protein symbol** | **Protein description** | **Log_2_FC** | **–Log_10_ (*p*-value)** |
| --- | --- | --- | --- | --- |
| **Proteins detected in both groups** | | |  |  |
| P68035 | ACTC1 | Alpha cardiac muscle 1 | 0.7 | 2.8 |
| P31232 | TAGLN | Transgelin | 1.2 | 2.3 |
| P0C0S7 | H2AFZ | Histone H2A.Z | 0.8 | 2.7 |
| P14669 | ANXA3 | Annexin A3 | 2.2 | 2.4 |
| Q08290 | CNN1 | Calponin-1 | 1.5 | 2.2 |
| P60711 | ACTB | Cytoplasmic 1 | 0.5 | 2.1 |
| Q64122 | MYL9 | Myosin regulatory light polypeptide 9 | 0.7 | 2.0 |
| Q63862 | MYH11 | Myosin-11 | 2.1 | 1.8 |
| Q5XFX0 | TAGLN2 | Transgelin-2 | 0.7 | 1.8 |
| P07943 | AKR1B1 | Aldose reductase | 2.2 | 1.8 |
| Q4V8H8 | EHD2 | EH domain-containing protein 2 | 1.2 | 1.7 |
| Q9WVH8 | FBLN5 | Fibulin-5 | 2.7 | 1.6 |
| P37397 | CNN3 | Calponin-3 | 3.4 | 1.5 |
| Q8CGS5 | ELAC2 | Zinc phosphodiesterase ELAC protein 2 | 0.6 | 1.5 |
| Q6VBQ5 | MYADM | Myeloid-associated differentiation marker | 0.9 | 1.5 |
| P41350 | CAV1 | Caveolin-1 | 2.6 | 1.5 |
| D3ZHA0 | FLNC | Filamin-C | 1.7 | 1.5 |
| Q62935 | VWF | von Willebrand factor | 2.5 | 1.5 |
| P48675 | DES | Desmin | 1.2 | 1.4 |
| Q9JLT0 | MYH10 | Myosin-10 | 2.1 | 1.3 |
| **Proteins detected only in UMHPAs** | | | | |
| P36201 | CRIP2 | Cysteine-rich protein 2 | | |
| P05982 | NQO1 | NAD(P)H dehydrogenase 1 | | |
| P12075 | COX5B | Cytochrome c oxidase subunit 5B | | |
| P16036 | SLC25A3 | Phosphate carrier protein, mitochondrial | | |
| Q5RKI0 | WDR1 | WD repeat-containing protein 1 | | |
| Q99PD6 | TGFB1I1 | Transforming growth factor beta-1-induced transcript 1 protein | | |
| Q9Z1Z9 | PDLIM7 | PDZ and LIM domain protein 7 | | |
| Q9HB97 | PARVA | Alpha-parvin | | |
| P11442 | CLTC | Clathrin heavy chain 1 | | |
| P43278 | H1F0 | Histone H1.0 | | |
| O35568 | EFEMP | EGF-containing fibulin-like extracellular matrix protein 1 | | |

UMHPA, unobstructed pulmonary artery with medial hypertrophy

## Table S2. Upregulated proteins in PLs.

| **Protein ID** | **Protein symbol** | **Protein description** | **Log_2_FC** | **–Log_10_ (*P*-value)** |
| --- | --- | --- | --- | --- |
| **Proteins detected in both groups** | | |  |  |
| P62329 | TMSB4 | Thymosin beta 4 | 1.5 | 3.4 |
| P08494 | MGP | Matrix Gla protein | 1.0 | 2.9 |
| Q5U206 | CALML3 | Calmodulin-like protein 3 | 1.7 | 2.8 |
| P08649 | C4 | Complement C4 | 1.9 | 2.1 |
| P62161 | CALM1 | Calmodulin | 0.5 | 2.1 |
| P10960 | PSAP | Sulfated glycoprotein 1 | 0.6 | 2.0 |
| B5DFC9 | NID2 | Nidogen-2 | 2.8 | 2.0 |
| **P04256** | **hnRNPA1** | **Heterogeneous nuclear ribonucleoprotein A1** | **1.5** | **1.9** |
| Q6B345 | S100A11 | Protein S100-A11 | 2.1 | 1.7 |
| P62959 | HINT1 | Histidine triad nucleotide-binding protein 1 | 1.5 | 1.6 |
| P30904 | MIF | Macrophage migration inhibitory factor | 0.7 | 1.5 |
| P04550 | PTMS | Parathymosin | 0.9 | 1.4 |
| P35704 | PRDX2 | Peroxiredoxin 2 | 1.5 | 1.3 |
| P01026 | C3 | Complement C3 | 2.9 | 1.3 |
| **Proteins detected only in PLs** | | | | |
| P14841 | CST3 | Cystatin-C | | |
| P55053 | FABP5 | Fatty acid-binding protein, epidermal | | |
| P21670 | PSMA4 | Proteasome subunit alpha type-4 | | |
| P02634 | S100G | Protein S100-G | | |
| P08427 | SFTPA1 | Pulmonary surfactant-associated protein A | | |
| Q7M767 | UBE2V2 | Ubiquitin-conjugating enzyme E2 variant 2 | | |
| Q64716 | INSRR | Insulin receptor-related protein | | |
| Q6IMF1 | KRT80 | Keratin, type II cytoskeletal 80 | | |
| P40307 | PSMB2 | Proteasome subunit beta type-2 | | |
| Q4KLN8 | RNF8 | E3 ubiquitin-protein ligase RNF8 | | |
| Q9QWN8 | SPTBN2 | Spectrin beta chain, non-erythrocytic 2 | | |
| Q62703 | RCN2 | Reticulocalbin 2 | | |
| Q05175 | BASP1 | Brain acid soluble protein 1 | | |

PL = plexiform lesion.
